# Transmission and Profile of COVID-19 in Children in North Sumatera, Indonesia

**DOI:** 10.34172/aim.2022.116

**Published:** 2022-11-01

**Authors:** Ayodhia Pitaloka Pasaribu, Restuti Hidayani Saragih, Fahmi Fahmi, Syahril Pasaribu

**Affiliations:** ^1^Department of Child Health, Medical Faculty, Universitas Sumatera Utara, Medan, Indonesia; ^2^Emerging Infectious Diseases Team, North Sumatera COVID-19 Task Force, Medan, Indonesia; ^3^Department of Internal Medicine, Medical Faculty, Universitas Sumatera Utara, Medan, Indonesia; ^4^Department of Electrical Engineering, Engineering Faculty, Universitas Sumatera Utara, Medan, Indonesia

**Keywords:** Children, COVID-19, Indonesia, Profile, Transmission

## Abstract

**Background:**

In December 2019, a cluster of viral pneumonia cases, later identified as coronavirus disease 2019 (COVID-19), was first reported in Wuhan, China, and then continued to spread to other parts of the world. COVID-19 is thought to be more prevalent in adults than children; therefore, information about COVID-19 burden and characteristics in children is lacking.

**Methods:**

We gathered data on the profile and transmission in children with COVID-19 from data collected by the North Sumatera Provincial Health Office team. Data were presented as mean±SD and percentage. Statistical analysis was performed using STATA version 15.0.

**Results:**

From April to October 2020, there were 1125 confirmed COVID-19 cases in children in North Sumatera, representing approximately 8.9% of all cases. Death occurred in 0.62% of the patients, and the children who died had underlying diseases. Four major clusters of COVID-19 infection in children were found in three Islamic boarding schools and one refugee shelter.

**Conclusion:**

A high number of children in North Sumatera were affected by COVID-19, and mortality was found to be higher in children with underlying diseases. Major clusters were found in places with prolonged and repeated activities in close contact, such as boarding schools and a refugee shelter.

## Introduction

 Since December 2019, coronavirus disease 2019 (COVID-19) has been a major public health concern worldwide.^1^ The first reported case was from the Huanan wholesale market in China, and then the disease spread rapidly to many countries across six continents.^2^ By November 24, there had been 58 712 326 confirmed cases, including 1,388,528 deaths worldwide due to COVID-19.^3^ The first case from Indonesia was reported on March 2, 2020. By the end of November 2020, there had been 506,302 confirmed cases in Indonesia with 3.2% of the total cases resulting in death.^4^ The main route of human-to-human transmission of COVID-19 is via droplets and contact with others.^5,6^ Transmission is thought to occur easily through close contact, such as between family members.^7^ The role of children in COVID-19 transmission is not certain.^8^ Children are also susceptible to SARS-­CoV-­2 infection but mostly have insignificant manifestations, raising the question of whether they play a role in the transmission of the virus or only suffer from the disease.^9^ In this study, we explored the profile and transmission in children with COVID-19 in North Sumatera, Indonesia. It is important to understand the burden of this disease in children to provide information for better prevention and early detection strategies to slow transmission in a community.

## Materials and Methods

###  Study Design and Participants 

 This was an existing data study evaluating the epidemiology and transmission of COVID-19 in children in North Sumatera. North Sumatera is the fourth largest city in Indonesia, consisting of 33 regencies/cities, with a total population of 14 562 549. The first confirmed adult case of COVID-19 in North Sumatera occurred in March 2020, followed by the first case of COVID-19 in a child in April. By the end of October 2020, the total number of COVID-19 cases in North Sumatera was 13,107, and North Sumatera was one of the national priorities for the management of COVID-19.

###  Data Collection

 Data on children with COVID-19 were extracted from all COVID-19 data that were collected by the North Sumatera Provincial Health Office team. The records included information on demographics, patient addresses, outcomes of patients and results of contact tracing. We defined a COVID-19 case as a person with a positive result on real-time reverse transcription-polymerase chain reaction (RT-PCR), according to the Indonesian Ministry of Health guidelines. Contact tracing was defined as the identification of persons who may have come into contact with a person with COVID-19 within 14 days prior to infection. An index case was defined as the first documented patient who contracted COVID-19 within a certain household or community. No identifying information, such as name, date of birth, or registration number, was collected.

###  Statistical Analysis 

 Descriptive statistics were used for all study variables. We presented continuous variables as mean ± SD and categorical variables as numbers (%). Statistical analyses were performed using STATA version 15.0.

## Results

 From April to October 2020, 1125 children were confirmed to have COVID-19 in North Sumatera. Data on patient characteristics are summarized in Table 1. Approximately 51.38% of the children who contracted COVID-19 were male, and 60.44% of the children were older than 10 years. However, 55 (4.89%) children under 1 year of age were diagnosed with COVID-19, and 6 of them were babies less than 2 weeks old. Four children had underlying diseases: two had leukemia, one had cardiovascular disease, and one was a premature baby. Seven children died (0.62%), four of whom were children with underlying diseases.

**Table 1:** Profile of Children with COVID-19 in North Sumatera from April to October 2020

**Characteristics**	**Number of Cases**	**Percent**
Gender		
Girl	547	48.62
Boy	578	51.38
Age (y)		
< 1	55	4.89
1-5	152	13.51
6-10	238	21.16
> 10	680	60.44
Underlying disease		
Leukemia	2	1.17
Cardiovascular disease	1	0.88
Premature birth	1	0.88
NA	3	0.27
Previously healthy	1118	99.37
Clinical outcome		
Recovery	957	85.07
Died	7	0.62
NA	161	14.40

NA, not available data.

Table 2 describes the details of death in children with COVID-19. There was no information on what caused death in cases no. 1, 4 and 5.

**Table 2:** Description of Death Cases in Children with COVID-19

**No.**	**Age**	**Gender**	**Residency**	**Underlying Disease**	**Condition at death**
1	2 months old	Boy	Deli Serdang	NA	NA
2	3 years old	Girl	Langkat	Leukemia	Pneumonia
3	8 years old	Boy	Simalungun	Leukemia	ARDS
4	7 years old	Boy	Karo	NA	NA
5	1 month old	Boy	Medan	NA	NA
6	1 month old	Girl	Medan	Cardiovascular disease	ARDS
7	Newborn	Girl	Medan	Premature	Respiratory distress

NA, not available data; ARDS, acute respiratory distress syndrome.

 Parents or siblings as the source of infection contributed to the highest number of COVID-19 cases in children (24.53%); mothers were the key source of infection for their children and attending a boarding school was also a significant contributor to contraction of COVID-19 (14.76%).

 Unfortunately, contact tracing was performed for only 46.13% of the cases. Only 12 children (1.07%) were the index case based on the reported data (Table 3).

**Table 3:** Transmission of COVID-19 in Children in North Sumatera.

**Transmission of COVID-19**	**Number of Cases**	**Percent**
Source of infection		
Parents or siblings	276	24.53
Relatives	16	1.42
Islamic boarding school	166	14.76
Refugee shelter	47	4.18
NA	620	55.11
Contact tracing performed		
Yes	519	46.13
No	606	53.87
Index case		
Yes	12	1.07
No	181	16.09
NA	932	82.84

NA, not available data

**Figure 1:**
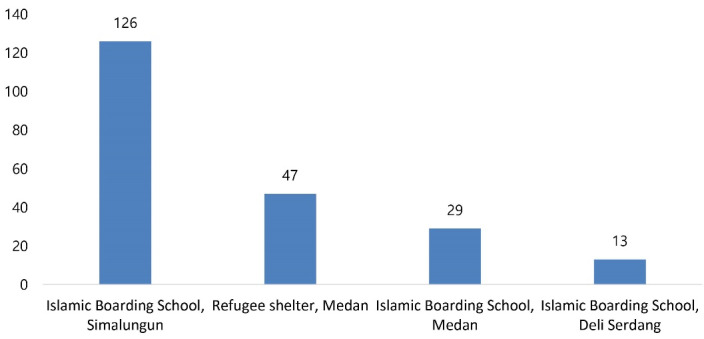
Major Clusters of COVID-19 in Children in North Sumatera

 Four major clusters of COVID-19 in children occurred, among which an Islamic Boarding School in Simalungun contributed to 126 cases in children, followed by a refugee shelter in Medan that contributed to 47 cases (Figure 1).

## Discussion

 North Sumatera is one of the largest provinces in Indonesia and one of the national priority targets for the management of COVID-19 due to the high number of cases in the province. The first case of COVID-19 in a child from North Sumatera occurred in April 2020, 1 month after the first case in an adult. The data in this study were collected between April and October 2020, and we found 1125 children with confirmed cases of COVID-19. The number of pediatric cases was 8.6% of the total number of COVID-19 cases in North Sumatera. This number was higher than that of pediatric cases in Italy (1.2%) or in the United States of America (5%).^10,11^ In our study, we found more cases in males (51.38%) than females (48.62%), which is similar to studies in China and Europe.^12,13^

 A study in China found that children aged 6-10 years were affected the most by COVID-19 (24.5%), while in Europe, the most affected age was < 2 years.^12,13^ These findings were in contrast to our result, where children > 10 years of age were the most affected in North Sumatera (60.44%). Among all children aged < 1 year, we found that 6 (10.9%) children with COVID-19 were less than 1 month old, 5 babies were under 1-week-old and 1 baby was 13 days old. Data from across Europe found that 7% of babies less than 1 month old had COVID-19.^13^ However, we found that children of all ages were as susceptible as adults to COVID-19, with ages ranging from 1 day to 17 years.

 We found two patients with COVID-19 who also had leukemia as an underlying disease, in addition to one patient who also had cardiovascular disease and one confirmed COVID-19 patient who was a premature baby. A study in Hubei, China, found that cardiovascular disease was an underlying condition in two critical patients; this was also confirmed by data from Europe, where 4% of the confirmed COVID-19 patients had cardiovascular diseases as an underlying condition and 5% of these cases had malignancy.^13,14^ Although only three children had underlying diseases, our study confirmed that these conditions could cause severe manifestations of COVID-19, as explained by Hoang et al.^15^

 There is very little information about potential vertical transmission of COVID-19 during pregnancy. However, several studies described that vertical transmission could not be excluded.^16,17^ In our study, we found that one newborn was delivered by caesarean section from a COVID-19-confirmed mother. The baby was born prematurely, had respiratory distress and died a few hours after birth despite resuscitation. Swab tests were collected before the baby died, and 1 day after death, the baby was confirmed positive for SARS-CoV-2.

 We found that 7 (0.62%) children with COVID-19 died, of whom two had leukemia, one had cardiovascular disease, and one was a newborn baby. Three other children who died were 1 month old, 2 months old and 7 years old without further information on the cause of death. Death was found more often in our study than the studies conducted by Yasuhara et al^18^ and Hoang et al.^15^ This may be explained by the severe manifestation of the underlying diseases or delay in seeking treatment. Since the COVID-19 pandemic occurred, people were hesitant to go to the hospital because of misinformation that hospitals were the source of COVID-19 or the misinformation that any patients would be considered to have COVID-19 if they went to the hospital (personal communication).

 A study across Europe showed that the most common source of COVID-19 infection in children was their parents (56%), which is similar to a study by Zheng et al in Hubei, China.^13,14^ Children actually have less contact with the outside world than adults; therefore, familial cluster transmission was the most common source of infection in children.^19^ A study performed by Zheng et al found that 84% of the children had a contact history, and only four patients did not.^14^ This was similar to our data, with only 12 children as index cases. Even though children are mostly infected by their family members, they may also be the source of infection.^20^ As children can be a hidden source of transmission, continuous contact tracing should always be performed. Unfortunately, contact tracing was only performed in 46.13% of all the confirmed COVID-19 cases that we found from our existing data.

 Four major clusters of COVID-19 infection were identified in our study that involved three Islamic boarding schools and one refugee shelter. One cluster of boarding schools comprised 126 locally transmitted cases in children. Mass gatherings, such as schools, churches, and shelters, could be transmission settings for COVID-19.^21-23^ COVID-19 is largely transmitted by close contact, especially for prolonged periods and through repeated social interactions.^21^ Both boarding schools and the refugee shelter host prolonged and repeated activities with close contact that might increase the risk of transmission. To prevent transmission, children should always practice personal hygiene, implement physical distancing and wear masks properly.^19,24^

 There is a limitation to this study because we used existing data and unfortunately, the data on contact tracing were not complete. However, this was the first reported data on epidemiology and transmission in children from North Sumatera, Indonesia.

 In conclusion, children constituted nearly 9% of COVID-19 cases, with a mortality rate of 0.62%. The majority of the children who died from COVID-19 had underlying diseases such as leukemia or heart disease. Aside from parents, major clusters of COVID-19 infection in children were found in boarding schools and a refugee shelter. Continuous education on preventive measures for children is necessary to minimize the risk of COVID-19 transmission.
